# Supplementary material for: Identification of small-molecule ion channel modulators in C. elegans channelopathy models
Source: Nat Commun. 2018 Sep 26;9:3941. doi: 10.1038/s41467-018-06514-5 (PMC6158242; doi:10.1038/s41467-018-06514-5)
Supplement: Supplementary file 1 — Supplementary Information [file 41467_2018_6514_MOESM1_ESM.pdf]

Supplementary Information

Identification of small-molecule ion channel modulators in *C. elegans* channelopathy models

Jiang *et al*

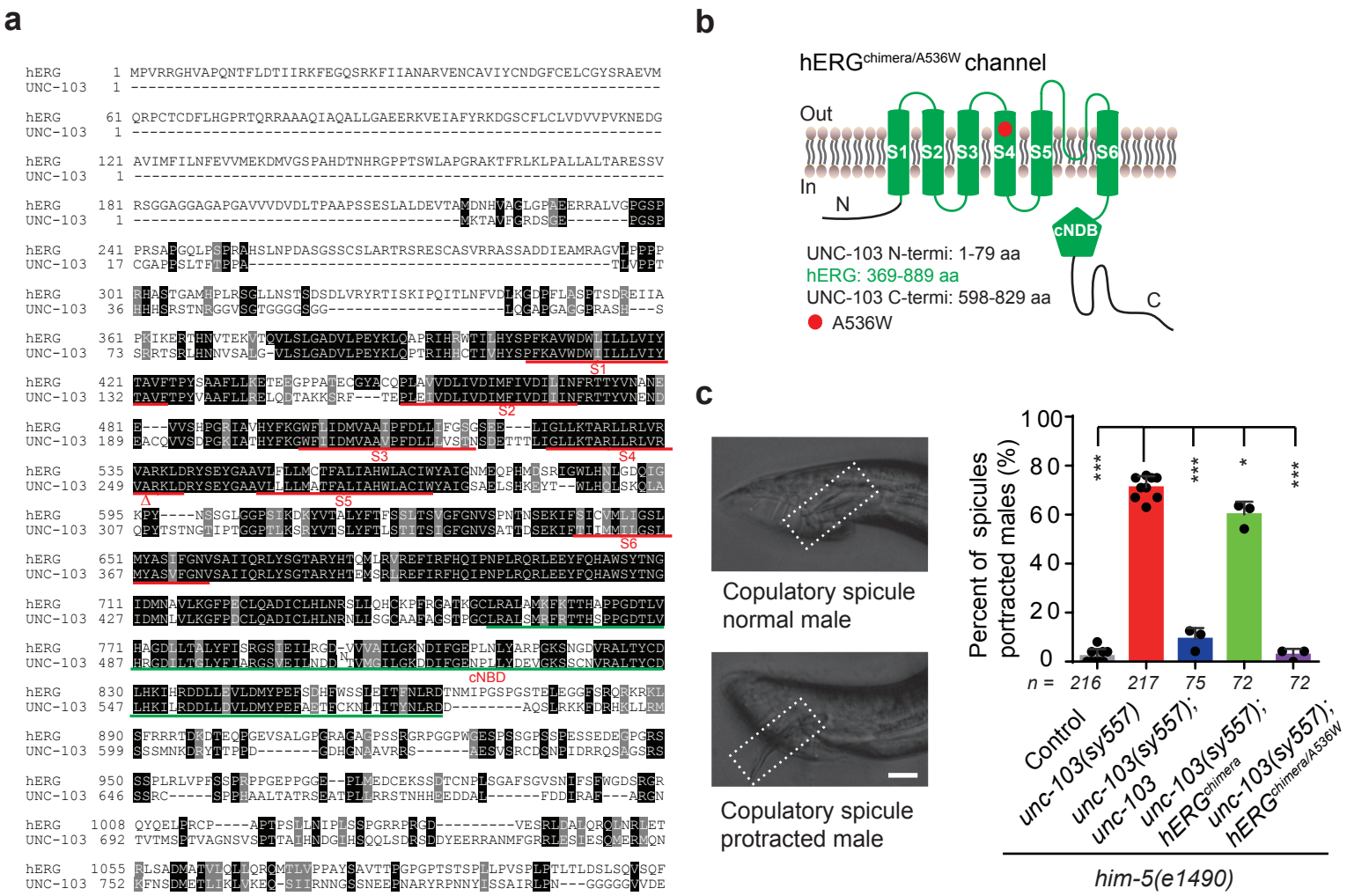

Supplementary Figure 1. hERG$^{chimera/A536W}$ rescued the copulatory spicule protraction defect of loss-of-function *unc-103* mutant worms. (**a**) Alignment of hERG and *C. elegans* UNC-103 K^+ channels. Transmembrane segments are underlined by red lines, and the red triangle shows the position of the gain of function mutation. cNBD, cyclic nucleotide-binding domain. (**b**) Schematic illumination of construction of hERG$^{chimera/A536W}$ channels. aa: amino acids. (**c**) Microscopic images of males with normal or protracted copulatory spicules (Left). Scale bar, 20 μM. Perecentage of males with protracted copulatory spicules (Right). Worms with *him-5(e1490)* background were used because they showed increased frequency of males in self-fertile populations. Data shown are mean ± s. e. m. $*P < 0.05$, $***P < 0.001$ (one-way ANOVA Dunnett's test). All experiments were performed at least three times.

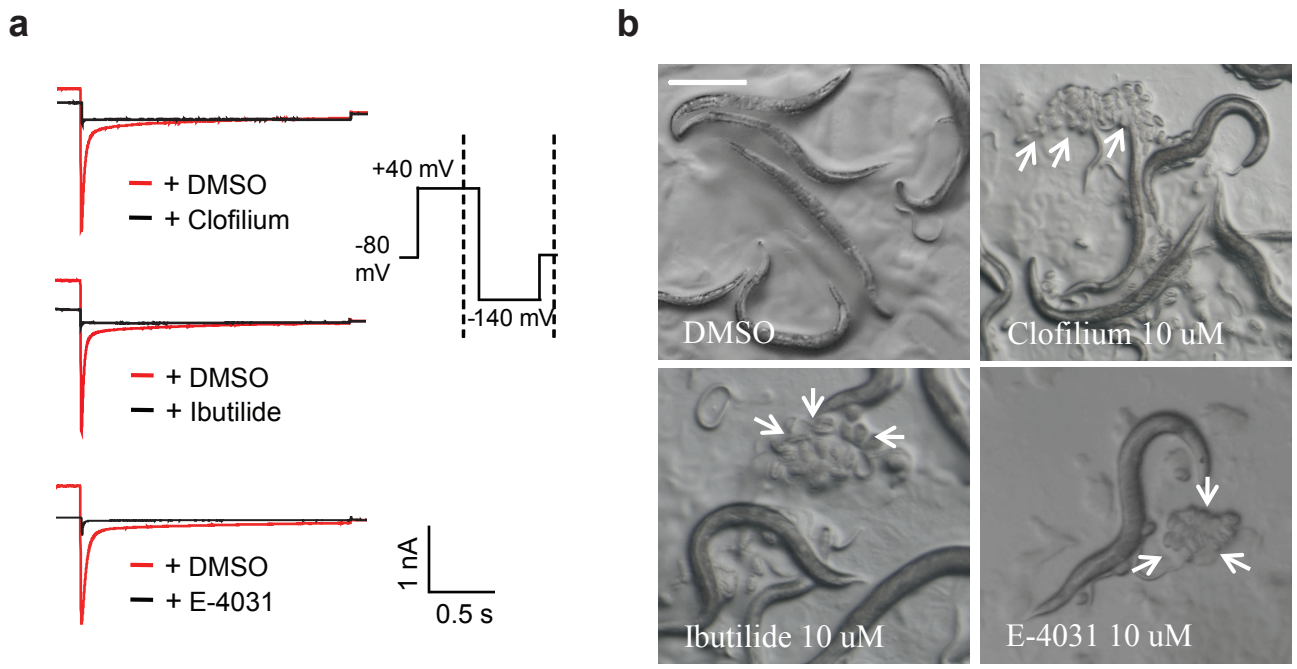

Supplementary Figure 2. hERG blockers ameliorate the phenotypic defects of *hERG*$^{chimera/A536W}$ transgenic worms. (**a**) Representative whole-cell currents of wild-type hERG channel expressed in HEK293T cells treated with DMSO or 1 μM hERG blockers Clofiliuim, Ibutilide, as well as E-4031. Protocol was shown in right upper, and traces at the time course between two dashes are shown. (**b**) Microscopic images of *acs-20;hERG*$^{chimera/A536W}$ transgenic worms in the absence or presence of 10 μM hERG blockers in cultivating plates. White arrows indicate eggs. Scale bar, 400 μm.

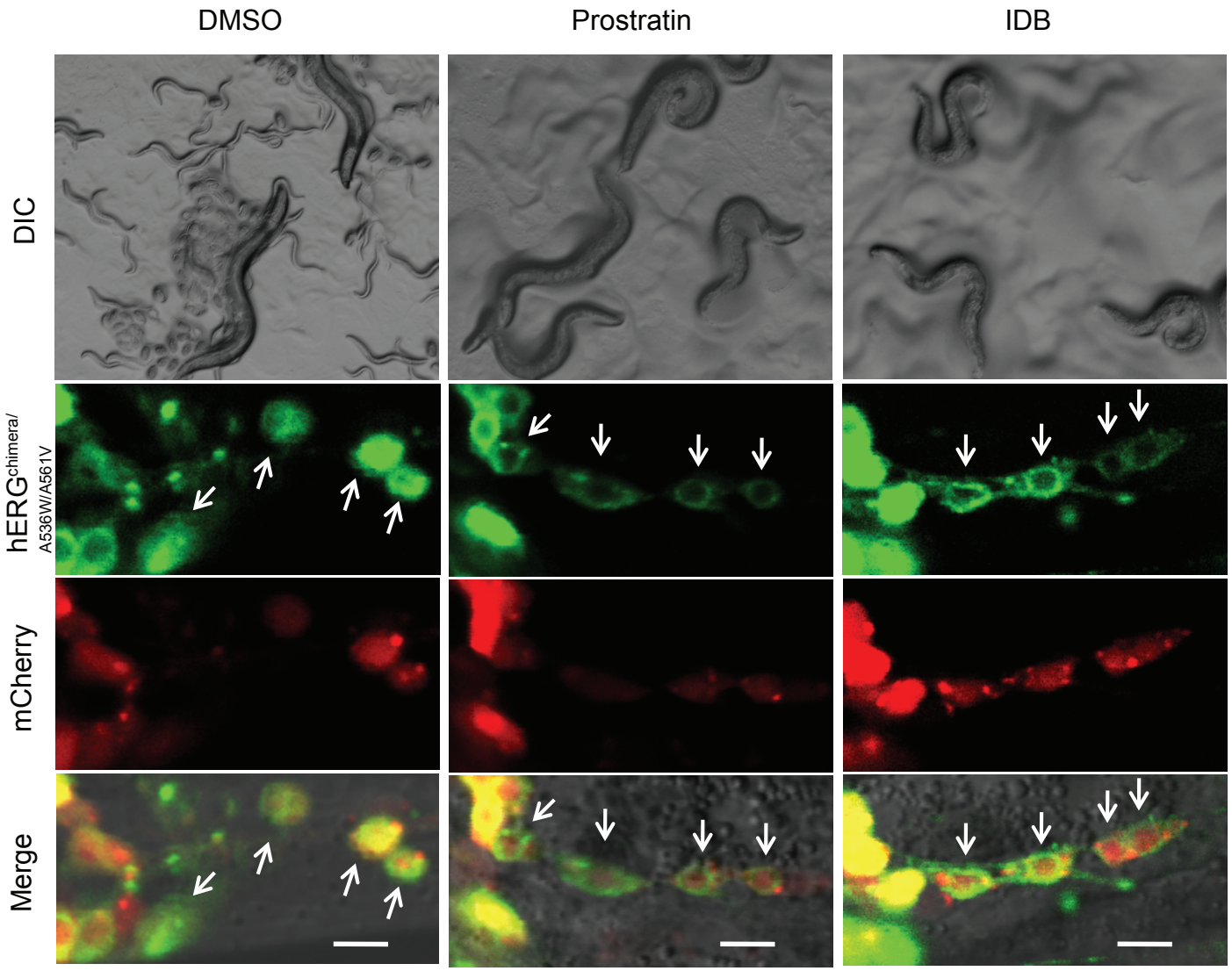

Supplementary Figure 3. Prostratin or IDB affects animal behaviors of hERG$^{chimera/A536W/A561V}$ transgenic worms and protein trafficking of hERG$^{chimera/A536W/A561V}$. Prostratin (20 μM) or IDB (20 μM) treatment induces behavioral defects and promotes trafficking of hERG$^{chimera/A536W/A561V}$::GFP in *hERG$^{chimera/A536W/A561V}$* transgenic worms. hERG$^{chimera/A536W/A561V}$::GFP and mCherry were driven by the *unc-103* promoter. Scale bar, 5 μm.

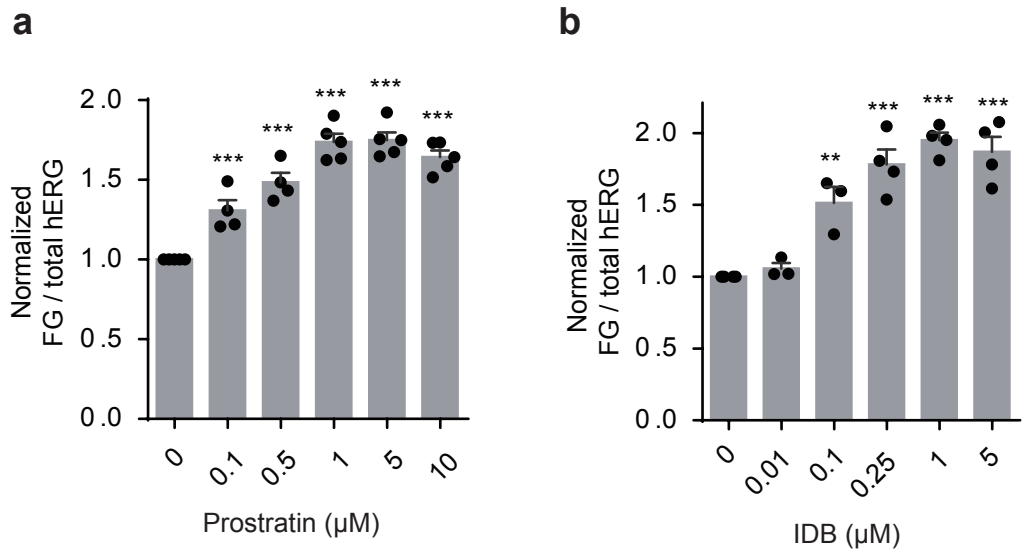

Supplementary Figure 4. Prostratin or IDB promotes trafficking of hERG^{WT}-hERG^{A561V} proteins. (**a-b**) Effects of Prostratin (**a**) and IDB (**b**) treatment on the ratio of fully glycosylated (FG) to total hERG^{WT}-hERG^{A561V} proteins expressed in HEK293T cells. The ratio of hERG^{WT}/hERG^{A561V} was 1:1 for **a**, **b**. Data shown are mean ± s. e. m. $^{**}P < 0.01$, $^{***}P < 0.001$ (one-way ANOVA Dunnett's test). All experiments were repeated at least 3 times.

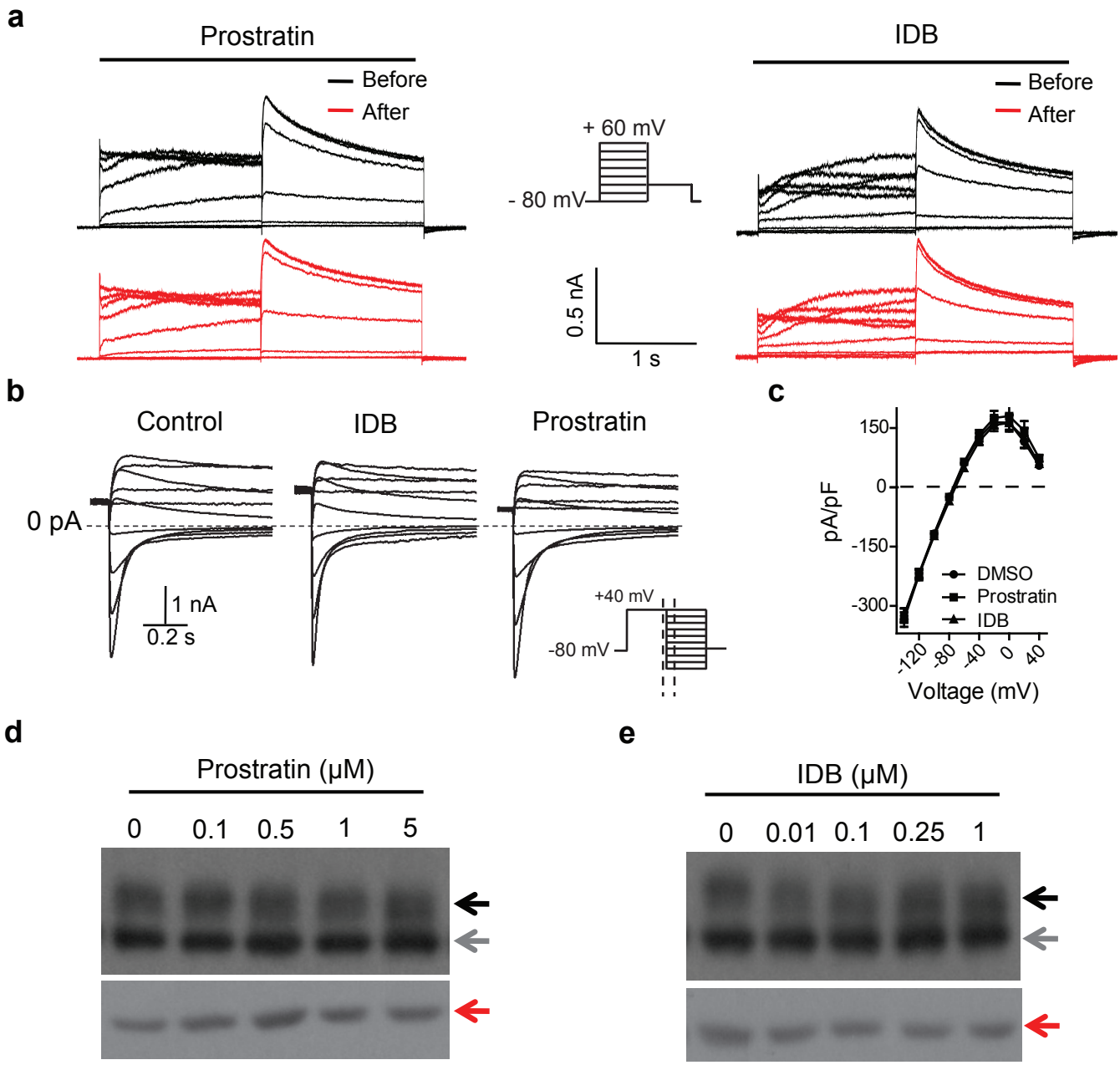

Supplementary Figure 5. Prostratin or IDB does not affect the function and protein trafficking of wild-type hERG K^+ channels in HEK293T cells. (**a**) Effects of acute Prostratin (3 μM, left) or IDB (2 μM, right) treatment on the function of wild-type hERG channels expressed in HEK293T cells. *n* = 6 cells for each treatment. Protocol was shown in the insert of the panel. (**b** and **c**) Effects of long-term Prostratin or IDB treatment on whole-cell recordings and the current density of hERG K^+ channels. *n* = 33, 26, and 32 cells for control, Prostratin and IDB treatment, respectively. (**d** and **e**) Effects of long-term Prostratin or IDB treatment on the protein trafficking of hERG channels. hERG proteins were visualized by anti-HA antibodies. Black, gray, and red arrows indicate 155 kD, 135 kD bands of hERG proteins, and tubulin, respectively. All experiments were repeated at least 3 times.

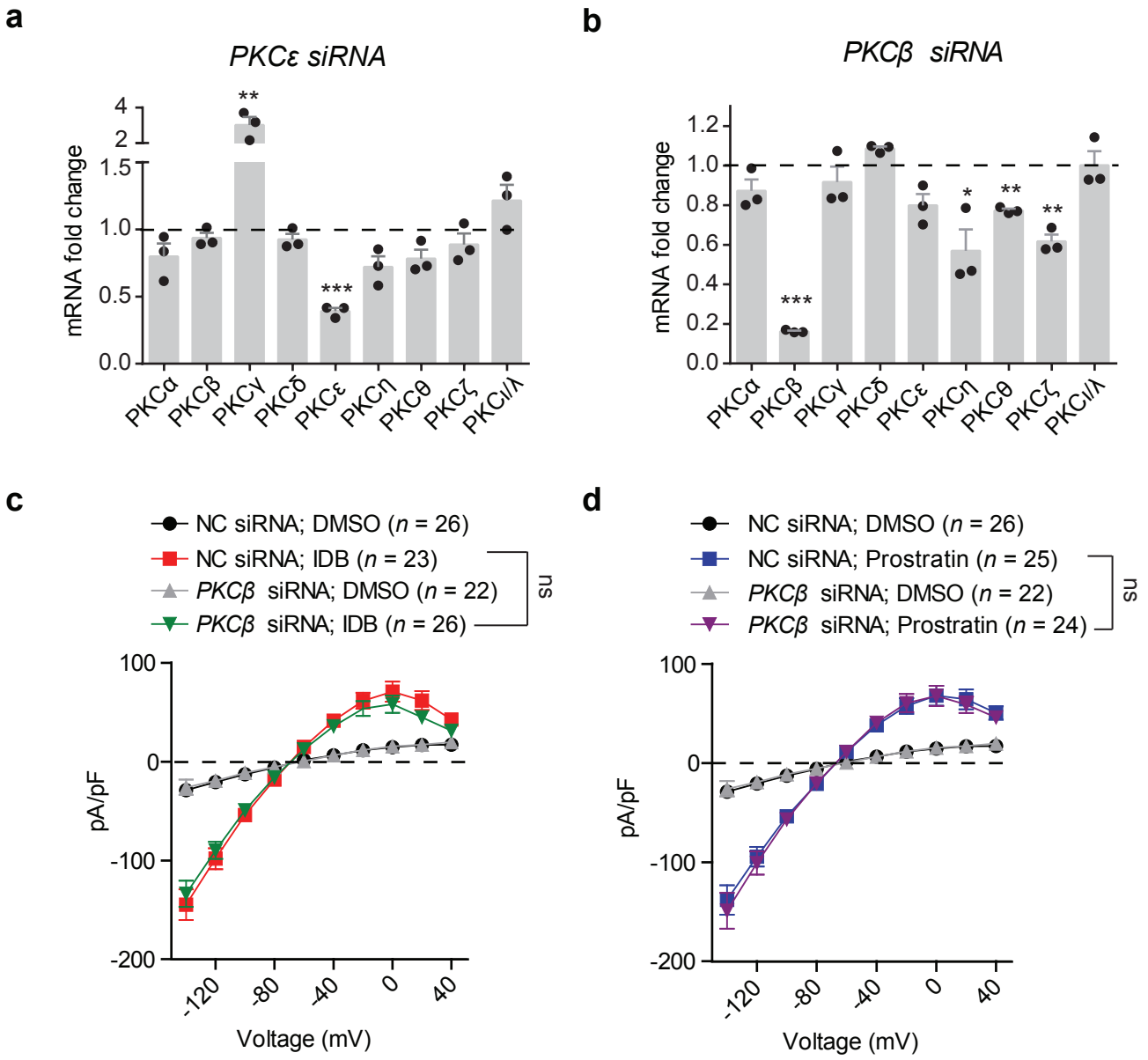

Supplementary Figure 6. Down-regulation of PKCβ does not affect the function of IDB and Prostratin on enhancing current density of hERG^{WT}-hERG^{A561V} channels. (**a** and **b**) Effect of *PKCε* or *PKCβ* siRNA on the transcription of isoforms of PKC proteins in HEK293T cells. (**c** and **d**) Effects of IDB and Prostratin on the function of hERG^{WT}-hERG^{A561V} channel expressed in HEK293T cells treated with control or *PKCβ* siRNAs. The ratio of hERG^{WT}/hERG^{A561V} was 2:1. All experiments were performed at least three times. Data shown are mean ± s. e. m. **P* < 0.05, ***P* < 0.01, ****P* < 0.001, ns: non significance (Student's t tests for **a**, **b**; one-way ANOVA Dunnett's test for **c**, **d**).

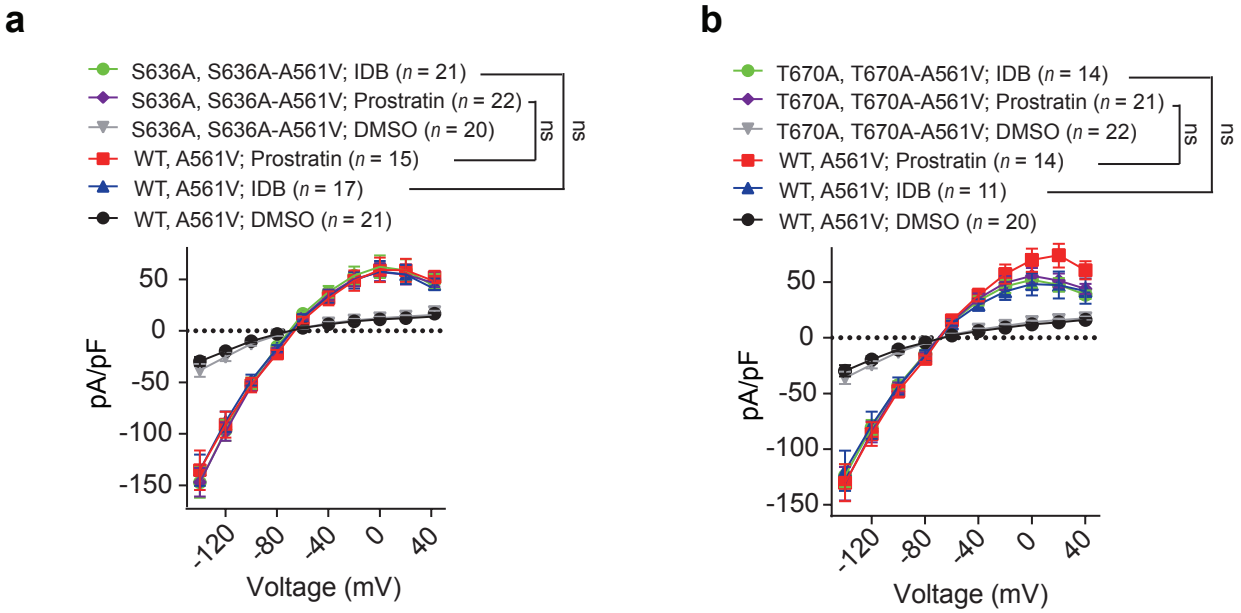

Supplementary Figure 7. Phosphorylation sites at S636 and T670 were not required for the function of IDB and Prostratin. (**a** and **b**) Effects of Prostrain and IDB on the current densities of $hERG^{WT}$-$hERG^{A561V}$ channels with a lack of the S636 or the T670 PKC phosphorylation sites. The ratio of $hERG^{WT}$/$hERG^{A561V}$ was 2:1**.** Data shown are mean ± s. e. m. ns, non significance (one-way ANOVA Dunnett's test). All experiments were performed at least three times.

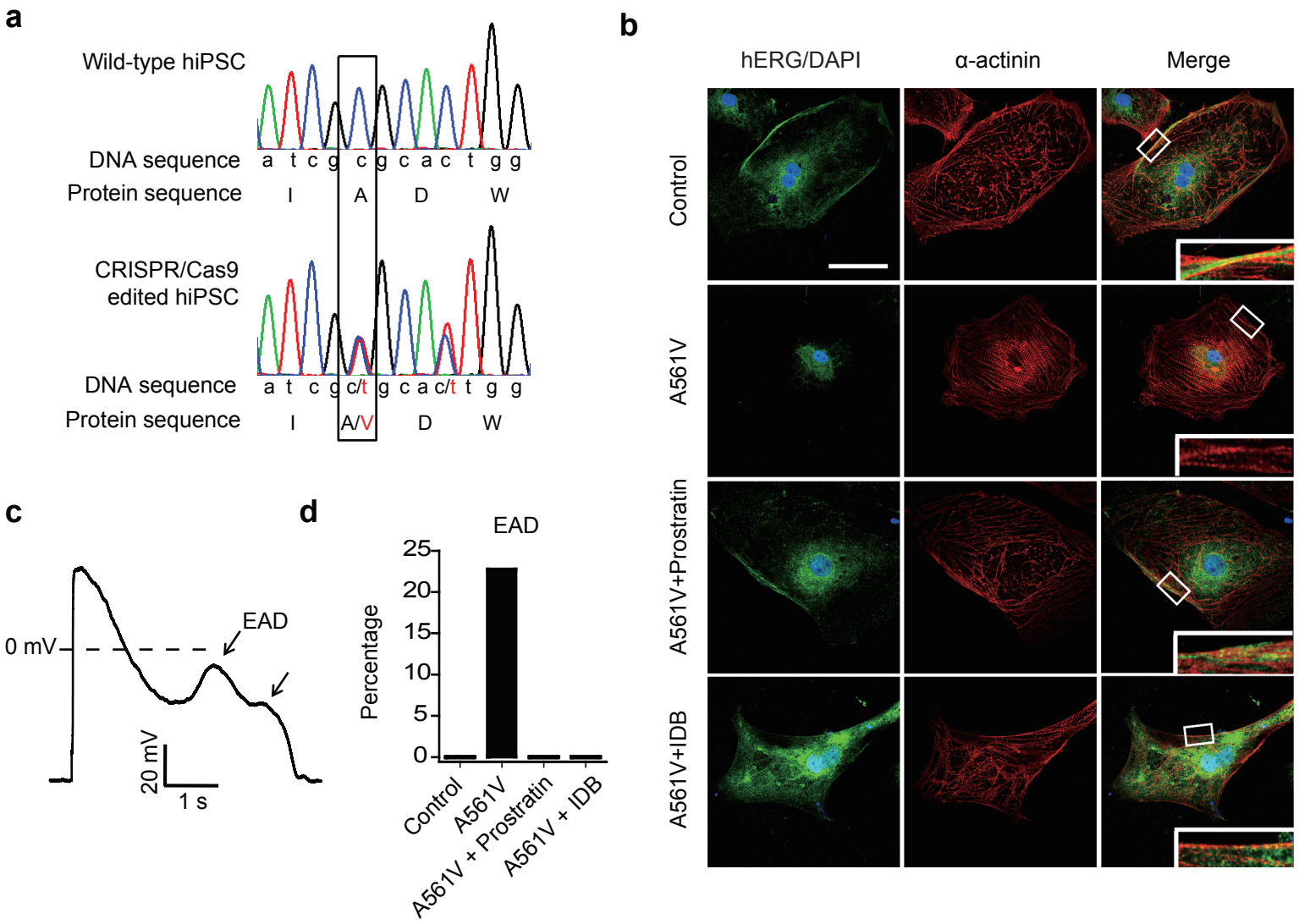

Supplementary Figure 8. Prostratin and IDB amelliorate the trafficking defect of hERG^{WT}-hERG^{A561V} proteins and prevent the incidence of EAD in hiPSC^{A561V}-CM. (**a**) DNA sequencing showing A561V heterozygous missense mutation made by CRISPR/Cas9-mediating genome editing in *KCNH2* locus of hiPSC. (**b**) Immunostaining for sarcomeric α-actinin and hERG proteins in control hiPSC-CMs or hiPSC^{A561V}-CMs treated with 3 μM Prostratin or 2 μM IDB. Scale bar: 50 μm. (**c**) Development of EAD in hiPSC^{A561V}-CMs. Arrows show early-after depolarization (EAD). (**d**) The effect of Prostratin (3 μM) or IDB (2 μM) on the incidence of EAD. n = 25, 22, 15 and 19 cells for control, A561V, A561V + Prostratin, and A561V + IDB, respectively. All experiments were repeated at least 3 times.

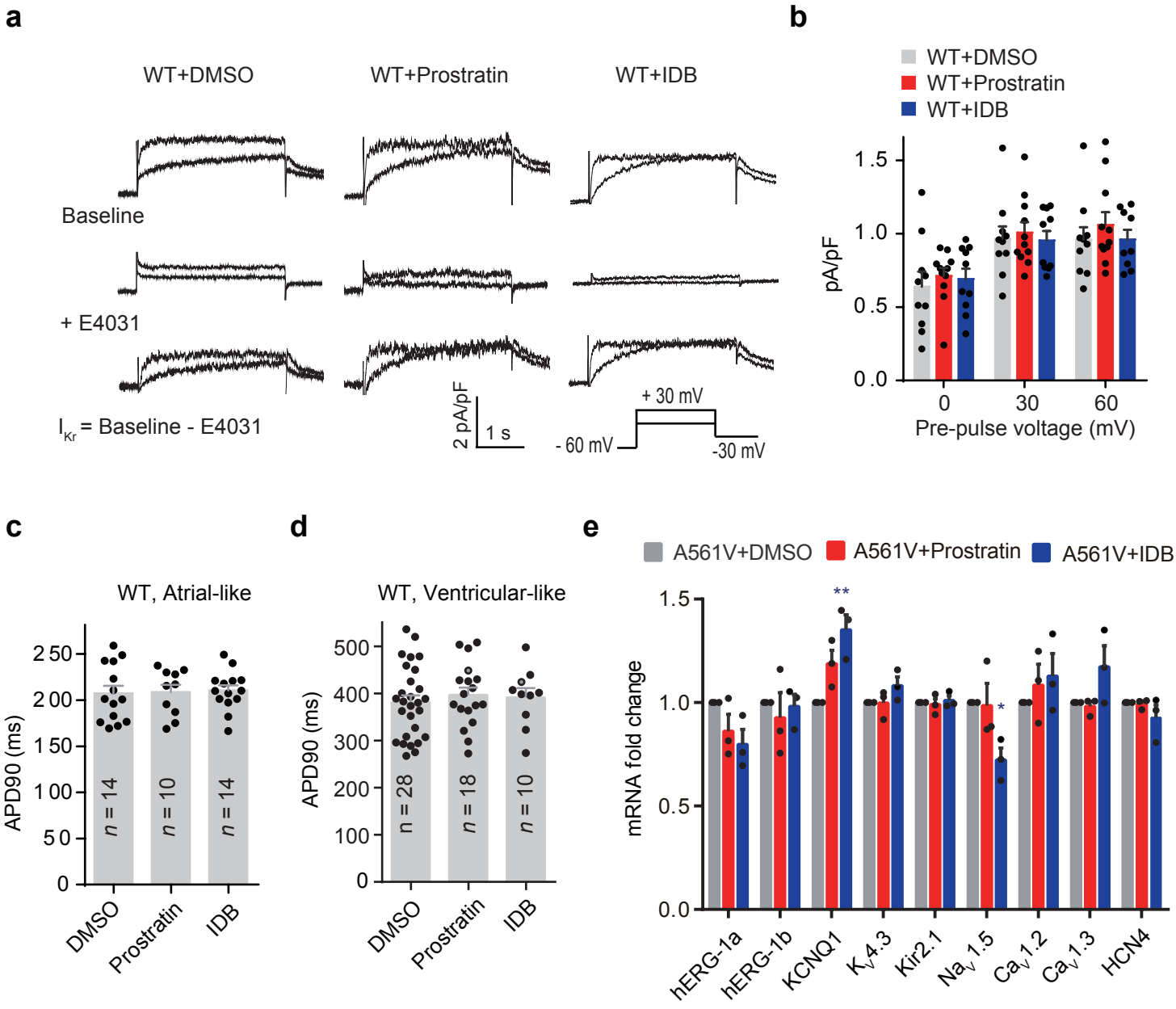

Supplementary Figure 9. Prostratin or IDB does not affect the wild-type I_{Kr} currents and and the durations of action potentials in hiPSC-CMs. (**a**) Representative I_{Kr} currents in hiPSC-CMs treated with DMSO, Prostratin (3 μM) or IDB (2 μM). (**b**) Tail current densities of I_{Kr}. *n* = 10, 11 and 10 cells for hiPSC-CMs + DMSO, hiPSC-CMs + Prostratin, and hiPSC-CMs + DB , respectively. (**c** and **d**) Effects of long-term treatment of Prostratin (3 μM) or IDB (2 μM) on the durations of action potentials in atrial-like and ventricular-like hiPSC-CMs; hiPSC-CMs used in this assay were purchased from Cellular Dynamics International. (**e**) Effect of Prostratin or IDB treatment on the mRNA levels of hERG splice variant and other ion channels in hiPSC^{A561V}-CMs. Data shown are mean ± s. e. m. **P* < 0.05, ***P* < 0.01, ns: non significance (one-way ANOVA Dunnett's test for **b**, **c**, **d** and **e**). All experiments were repeated at least 3 times.

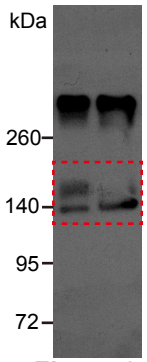

Figure 2g
(anti-HA blot)

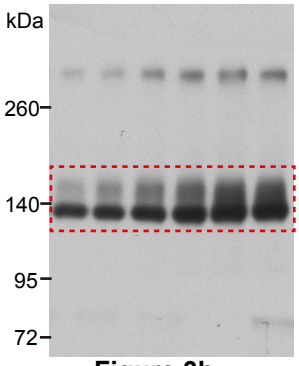

Figure 3h
(Left panel, anti-HA blot)

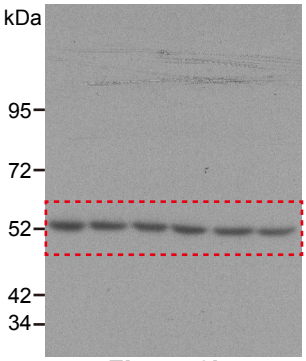

Figure 3h
(Left panel, anti-tubulin blot)

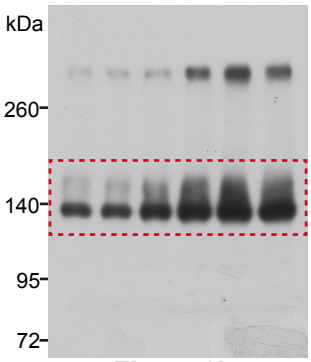

Figure 3h
(Right panel, anti-HA blot)

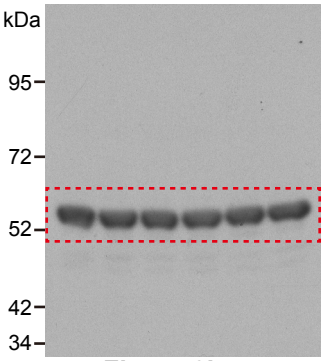

Figure 3h
(Right panel, anti-tubulin blot)

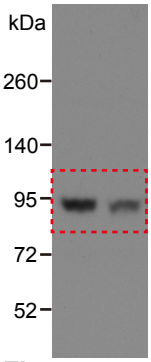

Figure 4a
(Top panel, anti-PKCε blot)

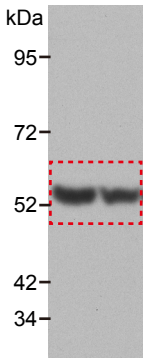

Figure 4a
(Down panel, anti-tubulin blot)

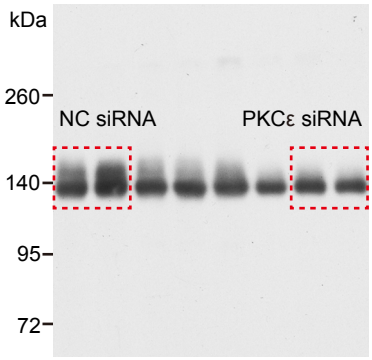

Figure 4d
(Left panel, anti-HA blot)

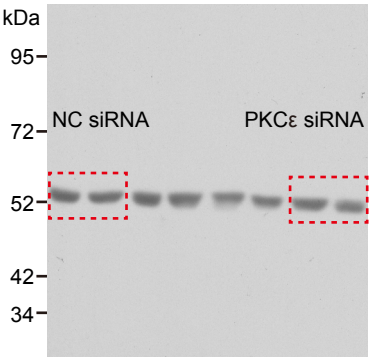

Figure 4d
(Left panel, anti-tubulin blot)

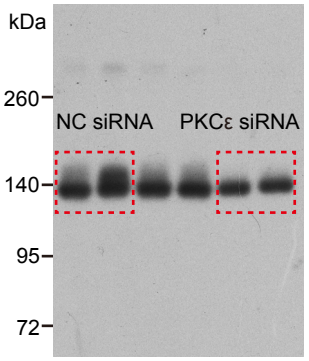

Figure 4d
(Right panel, anti-HA blot)

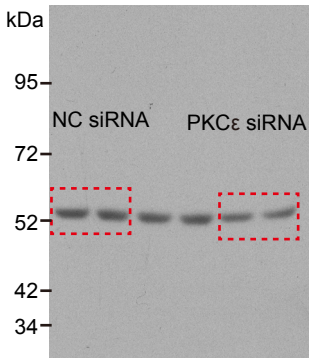

Figure 4d
(Right panel, anti-tubulin blot)

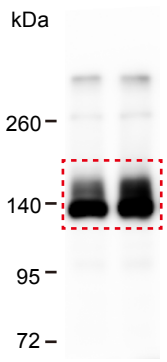

Figure 4f
(Top panel, anti-HA blot)

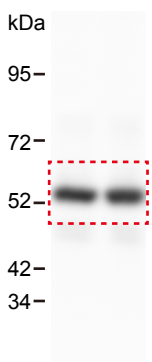

Figure 4f
(Down panel, anti-tubulin blot)

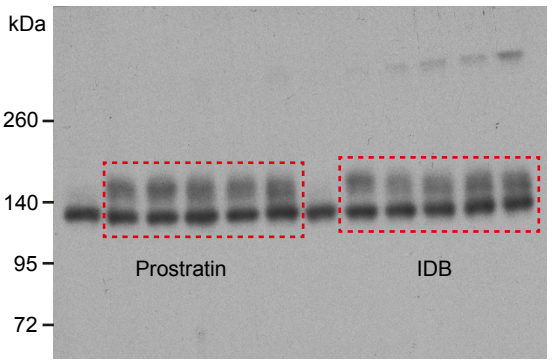

Supplemrnt Figure 5d, 5e
(Top panel,anti-HA blot)

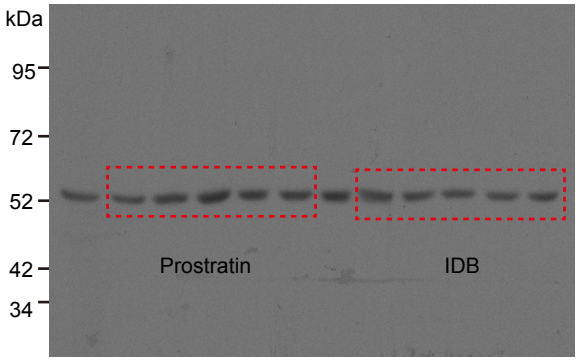

Supplemrnt Figure 5d, 5e
(Down panel,anti-tubulin blot)

Supplementary Figure 10. Full, uncropped images of immunoblots presented in the main text. Cropped areas are indicated by red boxes.
