# Supplementary material for: Identification of small-molecule ion channel modulators in C. elegans channelopathy models
Source: Nat Commun. 2018 Sep 26;9:3941. doi: 10.1038/s41467-018-06514-5 (PMC6158242; doi:10.1038/s41467-018-06514-5)
Supplement: Supplementary file 3 — Description of Additional Supplementary Files [file 41467_2018_6514_MOESM3_ESM.pdf]

Description of Additional Supplementary Files

File Name: Supplementary Movie 1
Description: The phenotype of wild-type N2 worms.

File Name: Supplementary Movie 2
Description: The phenotype of *hERG*$^{chimera/A536W}$ transgenic worms.

File Name: Supplementary Movie 3
Description: The phenotype of *hERG*$^{chimera/A536W/A561V}$ transgenic worms.
